# Malaria in Meghalaya: a systematic literature review and analysis of data from the National Vector-Borne Disease Control Programme

**DOI:** 10.1186/s12936-018-2563-3

**Published:** 2018-11-06

**Authors:** Anne Kessler, Anna Maria van Eijk, Limalemla Jamir, Catherine Walton, Jane M. Carlton, Sandra Albert

**Affiliations:** 1 Center for Genomics and Systems Biology, Department of Biology, New York University, New York, NY USA; 2 Indian Institute of Public Health-Shillong, Lawmali, Shillong, Meghalaya India; 3 University of Manchester, Manchester, UK; 4 Martin Luther Christian University, Shillong, Meghalaya India

**Keywords:** Malaria, Meghalaya, LLINs, Epidemiology, *Anopheles*, Complex malaria

## Abstract

**Background:**

Meghalaya, one of eight states in the northeastern region of India, has been reported to carry a high malaria burden. However, malaria surveillance, epidemiology, and vector studies are sparse, and no reviews combining these topics with malaria prevention and control strategies have been published in recent years. Furthermore, no analysis of surveillance data has been published documenting the changes in epidemiology following the first distribution of long-lasting insecticidal nets (LLINs) statewide in 2016.

**Methods:**

A hybrid approach was used to describe the status of malaria in Meghalaya. First, a literature search was performed using the terms ‘malaria’ and ‘Meghalaya’. Second, data were obtained from the Meghalaya State Malaria Control Programme for 2006–2017 for analysis of trends. Data from 3 years 2015–2017 were analysed further by district and year to assess changes in malaria incidence and distribution following the introduction of LLINs.

**Results/conclusions:**

Like malaria in mainland India, malaria in Meghalaya is complex, with both *Plasmodium falciparum* and *Plasmodium vivax* parasites in circulation, multiple *Anopheles* vector species, and reports of both unusual and severe malaria syndromes across all age groups. Integrated statewide malaria epidemiology, vector, and prevention and control data for Meghalaya are not readily available, and published studies are largely focused on a single topic or a single district or region of the state. Although malaria prevention and control approaches are available, (e.g. spraying, LLINs, personal repellents), their use and effectiveness is also not well characterized in the literature. Analysis of state malaria control programme data indicates that case incidence and related fatalities in Meghalaya have declined over the last decade. This could be attributed to changes in treatment guidelines and/or statewide distribution of effective prevention methods such as LLINs. Since the distribution of more than 900,000 LLINs in 2016, the malaria caseload has declined significantly in most Meghalaya districts, excluding the remote and geographically isolated South Garo Hills. Additionally, the proportion of adult malaria cases (15+ years of age versus children 0–14 years) in most districts was significantly greater following LLIN distribution, which likely reflects common lifestyle practices in these areas (e.g. adults working during night hours; small children in the households receiving priority for bed net protection). While reduction in malaria case incidence and related deaths is clear, the changes in malaria transmission and clinical manifestation have not been characterized. Routine epidemiology and vector surveillance combined with real-time data reporting are essential for the continued reduction and eventual elimination of malaria in Meghalaya.

## Background

Malaria has long been problematic in the mountainous state of Meghalaya [1], one of the eight northeastern states of India, known for their hilly, forested, and inaccessible regions, and for the indigenous/tribal people that live there. Although ‘indigenous peoples’ is a global term widely used in the literature, it is a contested term in India [2], and the Indian government instead uses the term ‘tribals’, or the constitutionally-recognized category of ‘Scheduled Tribes’ to refer to these communities in a countrywide sense [3]. Tribal communities in India are particularly prone to malaria because of their geographical marginalization, poor access to health care, low socio-economic status, and social factors including cultural and religious values, and beliefs [1, 4]. In Meghalaya, 86% of the state’s ~ 3.5 million people belong to scheduled tribes [5]. Most belong to the Khasi-Jaintia and/or Garo tribes, both of which are matrilineal, e.g., children take their mother’s name, and the youngest daughter inherits the ancestral land and looks after her parents in their elderly years. The state is divided into eleven districts: Garo Hills (North, East, West, South, South West), Khasi Hills (East, South West, West), Jaintia Hills (East, West), and Ri Bhoi, and each district houses a district hospital, community health centre/s (CHC), and primary health centres (PHCs) and sub-centres that service the inhabitants. Migration within and between the northeastern states is common and likely affects the malaria situation in Meghalaya.

Meghalaya is situated within the Indo-Burma biodiversity hotspot which spans from eastern Bangladesh through northeast India to Myanmar, southern China, Thailand, Lao PDR, Cambodia, Vietnam, the Andaman Islands, and Hainan [6]. The northeast comprise one of the distinct biogeographic regions of India with many endemic flora and fauna [7]. The very high rainfall of northeast India provides ideal conditions for growth of tropical forests that have historically covered the region. However, there has been substantial forest loss and fragmentation over recent decades in Meghalaya and the northeastern states of Tripura and Nagaland, primarily due to widespread shifting cultivation (‘*jhum*’) [8]. Although Meghalaya had 69% forest cover in 1999, a shift from closed to more open forest structure has occurred, particularly in the Khasi and Jaintia Hills [8]. Throughout much of Meghalaya, the forest cover forms a mosaic with a rice-agroecosystem such that villages in a malaria endemic area may be situated close to forests or rice fields or both. The diverse habitats this provides for multiple *Anopheles* species plays a large role in shaping the current and changing malaria epidemiology of this region.

Although malaria in India is a long-standing public health concern and a central focus of the National Vector Borne Disease Control Programme (NVBDCP), the government agency for the prevention and control of vector-borne diseases, there is a paucity of published data regarding malaria epidemiology, vectors, and control methods, and indeed health systems research in general [9], specific to Meghalaya. Over the past decade, malaria caused by the dominant species *Plasmodium falciparum* and *Plasmodium vivax* has been high and unstable across the state. From 2012, there was a steady increase in the number of malaria cases and deaths; however, cases have been decreasing since 2015. Meghalaya malaria control programme data suggests that the distribution of more than 900,000 long-lasting insecticide-treated bed nets (LLINs), distributed for the first time in 2016, could be responsible for the observed decline.

While malaria surveillance and reporting has been largely neglected across all eight Northeastern Indian states, the diversity and complexity of malaria as well as land cover, ecology, and host genetics of indigenous tribes specific to Meghalaya warrants independent assessment. Thus, the objectives of this review were twofold: first, to undertake a literature review of malaria in Meghalaya, with the goal of summarizing what is known about malaria epidemiology, vectors, and prevention methods in the state; and second, to undertake an analysis of statewide malaria epidemiology data from 2015 to 2017 to evaluate the effect of the introduction of LLINs. Malaria in Meghalaya is complex, with both *P. falciparum* and *P. vivax* prevalent, a plethora of key and emerging *Anopheles* vectors, severe and unusual clinical syndromes across age groups, and varied use and acceptance of different malaria prevention methods. Published studies focused on single districts have made generalization of malaria epidemiology across the state problematic; however in combination with analysis of Meghalaya State Malaria Control Programme surveillance they provide a solid foundation for continued malaria prevention, control, and elimination efforts.

## Methods

### Literature search strategy and selection criteria

To evaluate existing literature, PubMed and Google Scholar searches were performed using the search terms ‘Meghalaya’ and ‘malaria’. Studies were triaged for inclusion, and those which mentioned Meghalaya as a neighbouring state or case reports not relevant to malaria in Meghalaya were excluded. Case studies were excluded if the central focus was not a malaria diagnosis in Meghalaya. No limit on period of study was enforced when selecting papers for inclusion.

### Extraction and analysis of ‘Malaria in Meghalaya’ literature

Articles meeting the selection criteria were divided into three main categories: malaria epidemiology, malaria vectors, and malaria prevention and control. Within each category, sub-topics were identified and summarized as follows: malaria epidemiology: case incidence, distributions, disease presentations, fatality rates, and reporting; malaria vectors: key species, emerging species, vector diversity, and transmission factors; malaria prevention and control methods: use, effectiveness, and additional factors to consider for improved prevention. All papers selected for inclusion were read, summarized, classified by category, and discussed by at least two authors (AK and AVE).

### NVBDCP malaria surveillance data and analysis

**Fig. 1:**
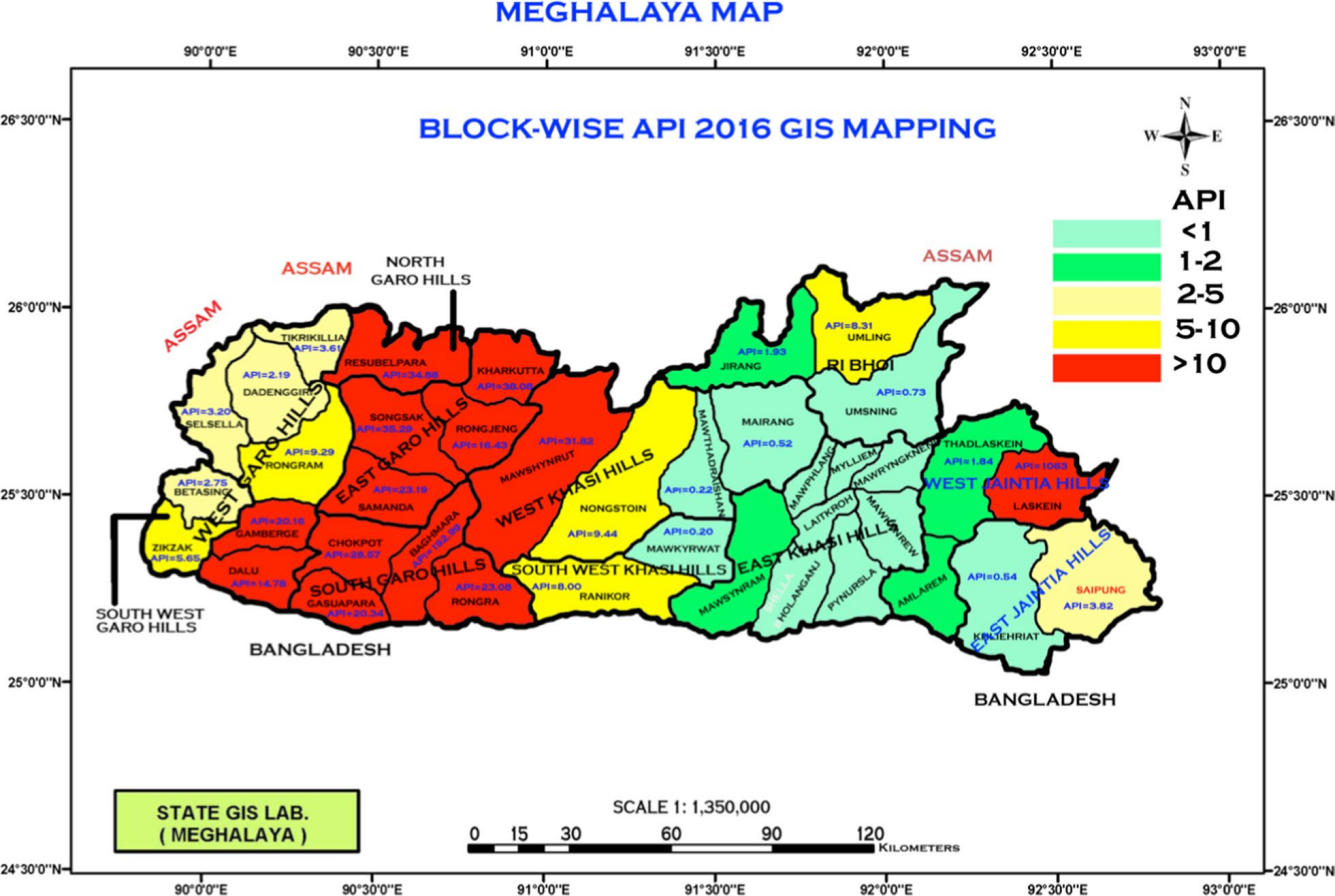
Burden and intensity of malaria in Meghalaya, 2016. Statewide depiction of block level malaria APIs during the year of LLIN distribution; District and block names are provided for reference, and API is indicated by both numeric value and color-coding where red indicates the highest malaria burden. Geographic borders, orientation, and scales are provided for completeness

District-wise epidemiology data from 2006 to 2017 were obtained from the Meghalaya NVBDCP and the primary health centres and sub-centres under their jurisdiction. Specifically, data detailing total malaria cases (both *P. falciparum* and *P. vivax*), malaria attributed deaths, and API was obtained for all districts. Age and sex distributions were available for malaria case and fatality data, while species-specific data were only available for the former. Timewise data from the last 3 years, 2015–2017, were obtained to assess and summarize the impact of LLINs distributed statewide for the first time in 2016. The statewide malaria burden and distribution in 2016 is provided for reference in Fig. 1. Information from East and West Jaintia Hills was combined for the analysis, and the data collected for East Khasi Hills were excluded because of the absence of malaria in most of the district, a result of the district’s high elevation. For all other districts, data from all PHCs and sub-centres was obtained and included in the analysis. The dataset was analysed in Stata 14.2, and summary figures were generated using Microsoft Excel. Statistical analysis was performed using Stata 14.2 or Prism 7.0. Statistical differences in clinical malaria by (1) age (15+ years versus children 0–14 years) and (2) sex (proportion of males) between the years preceding (e.g. 2015) and following (e.g. 2017) LLIN distribution were determined by Chi square tests. Statistical significance was assessed using an α level < 0.05.

## Results

**Fig. 2:**
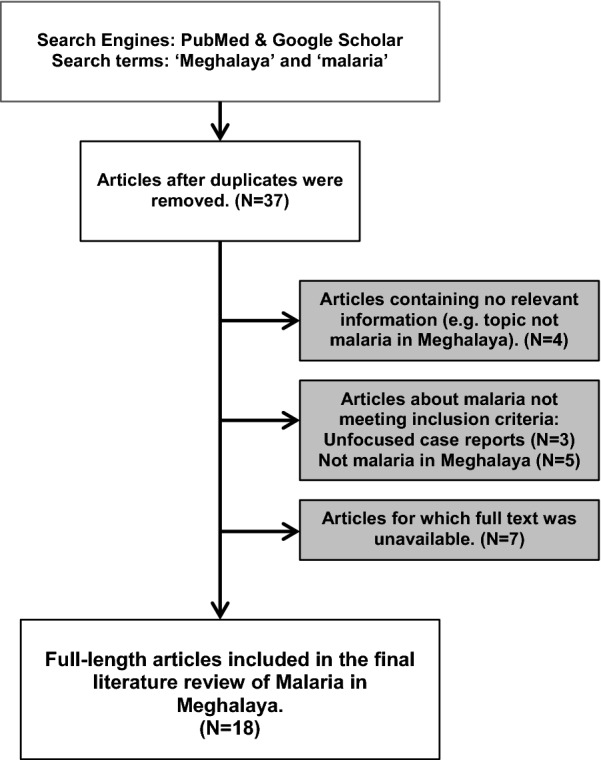
Selection process of malaria in Meghalaya articles for literature review

The literature searches in PubMed and Google Scholar yielded 37 articles (Fig. 2). Of these, 12 were immediately excluded for not meeting inclusion criteria. Specifically, four articles contained no relevant information (e.g., the topic of the article was not malaria in Meghalaya), three were case reports where malaria was not the central focus or diagnosis, and five were excluded for mentioning Meghalaya as a neighboring state, country, or another state on the Indian subcontinent. Additionally, the full text of seven articles could not be accessed (either in the USA or in India), and these articles were also excluded. In total, 18 articles focused on malaria epidemiology, vectors, and/or malaria prevention and control measures were included in the final review (Fig. 2).

### Literature review of malaria epidemiology in Meghalaya

Published reports of malaria epidemiology in Meghalaya described malaria transmission as perennial and persistent across the state [10, 11] and at border regions [12]. A retrospective analysis of malaria cases from 2001 to 2009 showed seasonal spikes in malaria, concurrent with the rainy (‘monsoon’) season which peaks during the months of May–July [11] but can extend into September–November [10]. While both *P. falciparum* and *P. vivax* were found across the state, *P. falciparum* was described as predominant in most regions [11, 13]. A retrospective analysis of *P. vivax* across all northeastern states from 2008 to 2013 indicated that *P. vivax* comprises 3–8% of malaria cases in regions of Meghalaya, making it less of a contributor to the malaria burden there than in neighbouring Indian states [13]. The authors also conducted malaria surveys from 1991 to 2012 to identify differences in *P. vivax* infection rates by age group, and found higher rates of *P. vivax* in children (0–15 years old) than in adults (15+ years of age) [13]. Mixed-species infections have also been reported in Meghalaya [14].

The northeastern states of India bridge the mainland to regions of east and south-east Asia, providing an avenue for the spread of parasite drug resistance into India. Current treatment recommendations for *P. falciparum* infections in India are 3 days of artemisinin-based combination therapy (ACT) and a single dose of primaquine on the second day of treatment [15]. However, whereas artesunate and sulfadoxine–pyrimethamine (ASP) are used in mainland India, the current guidelines for the northeastern states—including Meghalaya—recommend artemether–lumefantrine (AL) for *P. falciparum* infections [15]. This change in treatment guidelines occurred in 2013 [16] following growing reports of failed ASP reported in numerous northeastern states, including Mizoram, Tripura, Assam, and Arunachal Pradesh [17, 18]. The literature search revealed documented cases of severe malaria in Meghalaya that were successfully resolved using artesunate [19], but the degree of drug resistance to ACT in the state remains largely undocumented. The recommended treatment for *P. vivax* infection across the whole of India is chloroquine [15], which until 2004 was used to treat *P. falciparum* infections before the emergence of resistance, documented in northeast India [10, 11], Meghalaya, and adjacent states [13].

Although the majority of documented malaria cases in Meghalaya in the last decade are uncomplicated (i.e., cause mild disease), severe clinical presentations involving multiple organs including the central nervous, renal, hepatic, gastrointestinal, and pancreatic systems, have been reported in children [14] and adults [19]. Children (ages 0–17 years) with severe and unusual malaria syndromes were identified through a retrospective review of children admitted to the pediatric intensive care unit at NEIGHRIMS hospital in Shillong between 2006 and 2009 [14]. Of the 162 malaria cases identified, 10% (n = 16) had unusual, complicated syndromes including seven cases of cerebral malaria and eleven mixed species infections [14]. At the same hospital, a more recent report of a 65 year old man presenting with compounding, severe malaria complications was described [19]. While malaria-associated fatalities are relatively infrequent in Meghalaya, they continue to be reported [10, 20].

West Garo Hills (WGH) is the western most district in Meghalaya and routinely reports the highest number of malaria cases (nearly 100% *P. falciparum*) and malaria deaths. In 2009 and 2010, 72 and 32 deaths respectively were reported in the district through routine monitoring. A survey of 32 WGH villages in 2010 using different modeling approaches and validation of cause of death by verbal autopsy (e.g., confirmation of symptoms experienced before fatal outcome) was performed to determine a more accurate mortality rate and estimate the degree of under-reporting [20]. The models predicted that degree of under-reporting was 7–12 times, and that the true number of malaria-associated deaths was of the same order of magnitude as the World Health Organization estimate that same year (2009 WHO estimate = 452 versus 72 reported) [20]. A retrospective review of malaria rates from 2001 to 2009 conducted in the same district also indicated an overwhelming contribution from *P. falciparum* (98% of confirmed positives; majority of cases for all months and years in the study period) relative to *P. vivax*, and a high annual parasite index (API, range 12.9–68.3) throughout the study period [11].

### Literature review of malaria vectors in Meghalaya

Similar to epidemiology studies in Meghalaya, publications concerning malaria vectors in the state were sparse, although some extrapolation can be made from general studies of the northeastern states. For example, in the northeastern states, 23 different *Anopheles* vectors have been reported, highlighting the complexity of malaria in this region as a whole [10]. In general, *Anopheles minimus* was described as the major vector of the northeast alongside *Anopheles dirus* and *Anopheles fluviatilis*, which are thought to play minor but consistent roles in transmission [10, 11]. A state-wide survey of larvae and adult mosquitos conducted in Meghalaya in the 1980s identified 42 species belonging to six genera of which *Anopheles* was dominant [21]. Between 2001 and 2009, a review of mosquito prevalence and characterization conducted via daytime indoor collection and nighttime human landing catches in West Garo Hills identified the presence of *An. minimus* (density per person hour = 3.18) and zoophilic *Anopheles aconitus* (DPPH = 3.12) in high numbers [11]. The authors reported *An. minimus* as the main vector in the district with a sporozoite positive rate of 2.3%; all other vectors were sporozoite negative [11]. *Anopheles minimus* peak biting activity occurred between midnight and 02:00 h [11].

Meghalaya’s *Anopheles* population is diverse and complex and contains other potential malaria vector species. A survey conducted in northeastern India that included 25 villages spanning East Khasi Hills and East Garo Hills identified an abundance of *Anopheles culicifacies* vectors (95.7% of specimens collected) in addition to *An. minimus* (2.2%), *An. fluviatilis* (1.9%), and *An. dirus* (0.2%) [22]. From the 1564 Meghalaya mosquito specimens collected, 63 were positive for *Plasmodium* by ELISA, and 61 of the infected mosquitoes were *An. culicifacies*, incriminating it as a major vector in this region [22]. Another study of 10 villages from two regions along the Assam–Meghalaya border, including the Khasi Hills region of Meghalaya, identified *Anopheles annularis* and *Anopheles philippinensis/nivipes* (also reported in a Indian-wide survey that included Meghalaya [23]), to be the predominant vector species, with per trap night densities (PTND) of 4 and 4.1 respectively relative to *An. minimus* (PTND = 1.4) [24]. While *An. annularis* has historically been viewed as zoophilic, the study by Dhiman et al. found that 21.1% of blood-fed females fed on human blood and reported an overall anthropophilic index of 17.6–23.8% between sites [24]. The study found only one *An. annularis* mosquito infected with *P. falciparum* (2.6% infection rate) and did not claim a role for the species in malaria transmission.

A study of *An. minimus* and *An. dirus* in July–October of 2004 collected using CDC light traps in Williamnagar, East Garo Hills, used PCR of the ITS2 marker gene to identify species within these species complexes. *An. minimus* species A and *An. dirus* species D, now formally named *An. minimus* and *Anopheles baimaii*, respectively, were identified in Meghalaya and in adjacent northeastern states as well [25]. Further evaluation of the *An. dirus* complex in the northeast region confirmed a dominant presence of *An. baimaii* at forest fringes in all seven states, although the authors reported a relatively lower abundance of this sub-species in Meghalaya (e.g. smaller proportion of the state’s total vector population) compared to neighboring states [26]. Additionally, a comprehensive review of > 8000 malaria vector specimens collected from seven different countries highlighted the presence and contribution of *An. baimaii* specifically in Meghalaya [27].

Of importance to understanding vector dynamics are forces such as changes in the environment and ecology and the emergence of insecticide resistance. With respect to the former, a study of Meghalaya-specific data comparing changes in rice cultivation with observed changes in API at the same location between 1972 and 1983 demonstrated a significant dependence of API on the magnitude of rice cultivation, a finding that likely has minimal effect on malaria transmission in regions of the state where the stream-breeding species *An. minimus* is the sole or dominant vector [28]. The study by Akhtar et al. also explored changes in environment and vector prevalence, highlighting changes in forest coverage and the emergence of a previously under-represented vector species (e.g., *An. culicifacies*) in the northeastern states. Regarding insecticide resistance in the northeastern states and Meghalaya specifically, *An. minimus* demonstrated susceptibility to both dichlorodiphenyltrichloroethane (DDT, distributed through for example indoor residual spraying, IRS) and malathion (used to treat bed nets) in studies from the early 2000s [10, 11]. The study conducted by Dhiman et al. in 2011 along the border with Assam found that *An. annularis* has demonstrated resistance to DDT and low-level resistance to deltamethrin [24].

More recently, attention has also been paid to weather patterns and/or landscape changes that may lead to elevated levels of mosquito breeding, increased malaria transmission, and subsequently a need for enhanced or re-oriented control and prevention strategies. Specifically, a study found that increased El Nino intensity and a high Oceanic Nino Index result in an increase in malaria cases in Meghalaya in a given, concurrent year [29]. In India, El Nino oscillations have a demonstrated association with monsoon and rainfall indicators, and the development of such weather patterns could inform state and local control efforts of the need for enhanced distribution and utilization of available prevention tools given the demonstrated association with increased malaria case incidence. In addition to changing weather and climate patterns, alterations in landscape and land use can yield mosquito breeding habitats in cities and regions previously unaffected by malaria (e.g., high altitudes). This is an important consideration for a state like Meghalaya that is hilly with many villages and towns residing at high altitudes. A study conducted between 2008 and 2011 assessed the association between land use and land cover (LULC), and vector density and diversity, to determine the impact of deforestation and urbanization on mosquito breeding habitats statewide [30]. The study found that *Anopheles* development, diversity, and density was higher in the 16 villages adjacent to ‘disturbed land’, including urban, highway, and cultivated areas, relative to the four villages in natural, undisturbed areas [30]. The study findings highlight that LULC i.e., urbanization and deforestation, require monitoring to ensure proper malaria control and prevention strategies are in place, specifically in regions that were previously unaffected.

### Literature review of malaria prevention and control methods in Meghalaya

There are different malaria prevention and control efforts underway in Meghalaya that stem primarily from government sources. Central to control efforts is IRS of insecticide, which in Meghalaya is DDT, and the distribution of insecticide-treated bed nets (ITNs). IRS in Meghalaya is performed two times per year with priority for primary health centres (PHCs) and villages with an API > 2 [10, 11]. According to the PHC and village level accredited social health activist (ASHA) records, IRS is performed both indoors and outdoors (e.g. on the external siding of homes and shops) to varying degrees, and although statewide data are not available, the acceptance rate of DDT is recognized to be low [31]. During disease outbreaks when there is a rise in malaria cases, focal spraying has also been reported [11]. ITNs (bed nets impregnated with pyrethroids such as deltamethrin), are distributed under the supervision of the state malaria control programme at the PHC level as directed by the central government [10]. More than 900,000 LLINs were distributed on a gradual, rolling basis statewide in Meghalaya for the first time in 2016, where PHCs harbouring villages with the highest API from the previous year(s) received priority. At the PHC level, LLINs were uniformly distributed to all households over a span of days and weeks to maximize per-person coverage (e.g., distributed based on the number and ages of individuals in each household). LLINs, when properly maintained, are effective for three calendar years. A second distribution of LLINs in Meghalaya is scheduled to occur in 2019, dependent on the supply from the central government and distribution through the state health infrastructure. To supplement the IRS and ITNs, health education and self-protection awareness programmes organized by government and non-government organizations are often conducted during the malaria season or during unexpected periods of high transmission intensity [11].

In addition to the government-distributed control measures, personal mosquito repellents—including coils, vaporizers, mild repellent creams, and mats—represent a $1.5 billion industry in India [32] and are available across the state of Meghalaya. Little research has been done to confirm the utilization and effectiveness of repellent products nationally, but one multi-site study in mainland India found that utilization varies widely by household and individual and is associated with higher socio-economic status and level of education [32]. With regards to effectiveness, the study found that the use of mosquito repellents was associated with absence of malaria in some of the participant groups, but overall no consistent observation or association was observed [32]. In Meghalaya, no studies have been conducted regarding the efficacy of personal protection methods. A survey of 200 households conducted in two neighborhoods in Shillong, the capital of Meghalaya, in 2013 indicated that 42% of households surveyed use mosquito coils in comparison to 3.9% that reported burning tactics (e.g. burning of leaves, wood, or cloth to generate smoke), 13% that indicated bed net use, and 41.1% that adopted no measures [31]. While informative, the study was conducted in an urban setting in East Khasi Hills, the district with the least malaria prevalence, and likely sampled a population of people living at a higher SES and with a higher education level than what would be expected statewide, specifically in the villages most affected by malaria.

Timely and effective treatment for clinical malaria is imperative to disease transmission and thus also key for malaria prevention and control. The survey by Battacharyya et al. [31] also evaluated treatment-seeking behaviour, revealing that 97% of those surveyed indicated that they sought treatment at a regulated facility (66.9% government hospitals and 30.1% private hospital/doctor) with only 3% reporting self-medication. Primary, facility-based treatment in Meghalaya involves both active detection (i.e., malaria clinics at the PHC or village level) and passive detection (i.e., symptomatic individuals come to the PHC) [10]. In addition to facility-administered treatment, alternative medicinal approaches have been reported across the northeastern states. Specifically, interviews with traditional healers and village elders indicated that people use > 65 different plants belonging to more than 38 plant families to treat malaria [33]. In Meghalaya, the use of roots, leaves, bark, whole plants (oral), root powder (oral), leaf oil (oral), and leaves (chewed with betel nut leaves) was reported for malaria treatment [33]. Crude preparations are typically prepared by boiling/decoction of plants or plant parts in water to ease ingestion [33]. Individuals who self-medicate use similar alternative approaches as opposed to the recommended, hospital administered drug treatment(s), most likely due to availability and cost.

### Analysis of Meghalaya State Malaria Control Programme 2015–2017 and the impact of LLINs

**Fig. 3:**
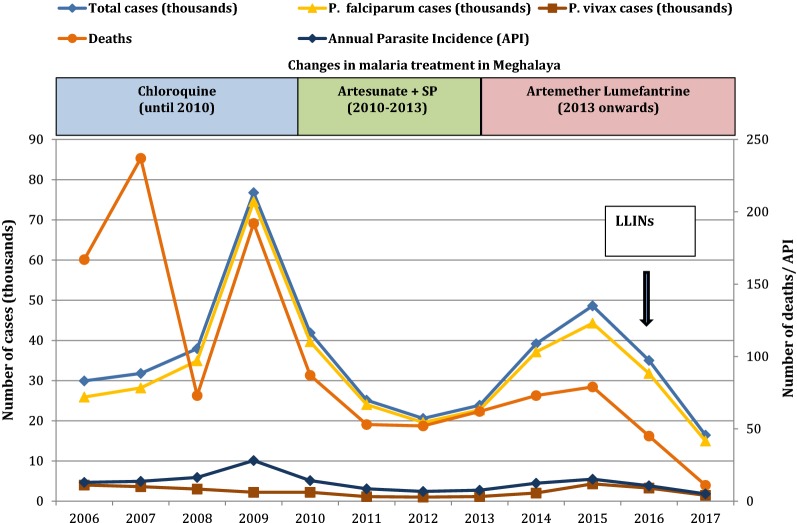
Statewide malaria situation in Meghalaya, 2006–2017. Anti-malarial drugs in use are shown as follows: CQ: chloroquine; AS + SP: artesunate and sulfadoxine/pyrimethamine; AL: artemether/lumefantrine. The initial distribution of LLINs is indicated with a black arrow

**Fig. 4:**
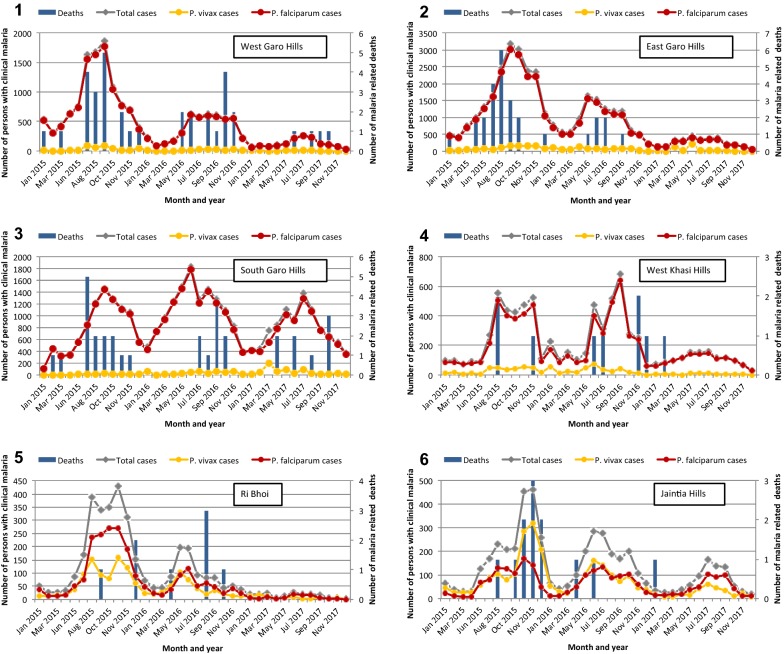
Monthly malaria cases in six districts in Meghalaya, 2015–2017. (1) West Garo Hills, (2) East Garo Hills, (3) South Garo Hills, (4) West Khasi Hills, (5) Ri Bhoi, (6) Jaintia Hills

At the state level, malaria cases and malaria-related deaths in Meghalaya have decreased over the last 11 years (Fig. 3). Changes in malaria policy, including the change from chloroquine treatment to antimalarial combination therapy and/or the statewide distribution of LLINs in 2016, indicated in Fig. 3, are most likely responsible for this observation. While continued surveillance and reporting is necessary to determine the true impact of the LLINs, the decline in malaria case incidence and fatalities during the distribution time frame is visually clear (Fig. 3). To assess the suspected impact of mass LLIN distribution in the state and improve understanding of the current malaria picture in Meghalaya, total malaria cases, species-specific malaria cases (*P. falciparum* and *P. vivax*), and malaria deaths were quantified and compared by district and year for 2015 (year preceding LLIN distribution), 2016 (year of LLIN distribution), and 2017 (year following LLIN distribution) in the seven malarious Meghalaya districts (note that East and West Jaintia Hills were combined for analysis). Summary data was first plotted to depict changes and trends over the time frame of interest. *P. falciparum* was found to be the dominant cause of malaria in four districts (≈ 90% or more of infections present in West Garo Hills, East Garo Hills, South Garo Hills, and West Khasi Hills), whereas *P. vivax* was more common in Jaintia Hills (≈ 50%) and Ri Bhoi (≈ 40%) (Fig. 4, panels 1–6). Although malaria cases and deaths were still reported in 2017, a steady decline in both malaria cases and deaths was observed in both West and East Garo Hills across the three-year time frame (Fig. 4, panels 1–2). In contrast, South Garo Hills and West Khasi Hills maintained comparable levels of both malaria cases and deaths from 2015 to 2017 and 2015–2016, respectively (Fig. 4, panels 3–4). In Ri Bhoi and Jaintia Hills, the two districts where *P. vivax* is present, malaria cases and deaths declined incrementally each year from 2015 to 2017, with cases and deaths dropping severely to single digit numbers in Ri Bhoi by 2017 (Fig. 4, panels 5–6). As outlined in the literature review results, malaria in Meghalaya is persistent and perennial but rises during the monsoon season and summer months of June to September. Despite the decline in overall and species-specific malaria cases, this cyclic trend was observed for total malaria cases, *P. falciparum* cases, and *P. vivax* cases (when present) across all 3 years (Fig. 4).

Key to understanding malaria epidemiology and changes in case incidence in a given region are age- and sex-specific infection distributions following a new intervention. In all six districts, the majority of malaria infections were diagnosed among persons ≥ 15 years (range 42–64%), with young children (0–4 years) contributing 12–25% of all infections, and older children (5–14 years) contributing 21–32% (Table 1). By gender and age in 2015, males dominated in the age group ≥ 15 years in West Garo Hills (55.7% males compared to 50.9% males in age group < 15 years, p < 0.001), West Khasi Hills (54.7% versus 49.8%, p = 0.006), Ri Bhoi (58.1% versus 52.8%, p = 0.009) and Jaintia Hills (59.1% versus 51.2%, p < 0.001), whereas females in the age group ≥ 15 years were more likely to have malaria in East (51.4% female among ≥ 15 years compared to 49.5% female in age group < 15 years, p = 0.007) and South Garo Hills (54.4% female among ≥ 15 years compared to 49.7% female in age group < 15 years, p < 0.001), the districts with the highest API (Table 1).

**Table 1:** Malaria cases within each district by age and sex, 2015–2017

District	West Garo Hills			East Garo Hills			South Garo Hills			West Khasi Hills			Ri bhoi			Jaintia Hills		
	M (row %)	F	All (col %)	M (row %)	F	All (col %)	M (row %)	F	All (col %)	M (row %)	F	All (col %)	M (row %)	F	All (col %)	M (row %)	F	All (col %)
2015 (years)																		
< 5	703 (49.8)	708	1411 (13.0)	2628 (50.3)	2594	5222 (26.1)	927 (50.5)	909	1836 (19.7)	319 (49.9)	320	639 (19.9)	217 (51.8)	202	419 (17.7)	242 (50.3)	239	481 (21.0)
5–14	1721 (51.4)	1626	3347 (30.9)	3234 (50.6)	3153	6387 (31.9)	1557 (50.2)	1543	3100 (33.2)	410 (49.7)	415	825 (25.7)	425 (53.3)	372	797 (33.6)	333 (52.0)	308	641 (28.0)
15+	3387 (55.7)	2693	6080 (56.1)	4096 (48.6)	4340	8436 (42.1)	2005 (45.6)	2394	4399 (47.1)	955 (54.7)	792	1747 (54.4)	670 (58.1)	483	1153 (48.7)	690 (59.1)	478	1168 (51.0)
	5811 (53.6)	5027	10838	9958 (49.7)	10087	20,045	4489 (48.1)	4846	9335	1684 (52.4)	1527	3211	1312 (55.4)	1057	2369	1265 (55.2)	1025	2290
2016 (years)																		
< 5	338 (53.4)	295	633 (13.5)	1356 (48.5)	1438	2794 (25.4)	1517 (47.4)	1682	3199 (24.4)	321 (51.4)	303	624 (18.9)	82 (45.8)	97	179 (17.3)	165 (54.1)	140	305 (17.5)
5–14	702 (52.5)	636	1338 (28.6)	1768 (49.3)	1815	3583 (32.5)	1978 (51.7)	1848	3826 (29.2)	414 (49.9)	416	830 (25.1)	180 (58.4)	128	308 (29.8)	247 (52.0)	228	475 (27.2)
15+	1579 (58.3)	1129	2708 (57.9)	2206 (47.6)	2427	4633 (42.1)	2726 (44.9)	3345	6071 (46.4)	963 (51.9)	891	1854 (56.0)	311 (57.1)	234	545 (52.8)	552 (57.1)	414	966 (55.3)
	2619 (56.0)	2060	4679	5330 (48.4)	5680	11,010	6221 (47.5)	6875	13,096	1698 (51.3)	1610	3308	573 (55.5)	459	1032	964	782	1746*
2017 (years)																		
< 5	89 (45.6)	106	195 (12.5)	343 (49.1)	356	699 (22.7)	1156 (49.5)	1181	2337 (24.9)	96 (50.0)	96	192 (15.1)	12 (57.1)	9	21 (11.3)	59 (50.0)	59	118 (14.5)
5–14	195 (53.0)	173	368 (23.5)	479 (51.5)	451	930 (30.2)	1279 (48.8)	1344	2623 (28.0)	134 (50.4)	132	266 (20.9)	33 (60.0)	22	55 (29.6)	100 (57.8)	73	173 (21.3)
15+	567 (56.7)	433	1000 (64.0)	648 (44.5)	807	1455 (47.2)	1884 (42.6)	2538	4422 (47.1)	418 (51.2)	398	816 (64.1)	61 (55.5)	49	110 (59.1)	304 (58.2)	218	522 (64.2)
	851 (54.4)	712	1563	1470 (47.7)	1614	3084	4319 (46.0)	5063	9382	648 (50.9)	626	1274	106 (57.0)	80	186	463	350	813

M: male, F: Female, row %: row percentage, col %: column percentage

* Age and gender was missing for 4 persons in Jaintia Hills

**Fig. 5:**
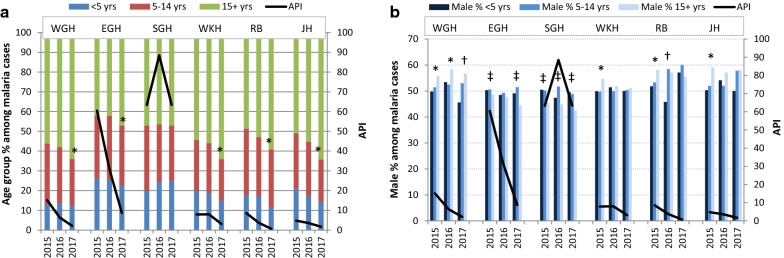
Clinical malaria by age and sex, 2015–2017. **a** Percentage of malaria cases (y-axis) for each age group (< 5 years, 5–14 years, 15+ years) in each district; WGH: West Garo Hills, EGH: East Garo Hills, SGH: South Garo Hills, WKH: West Khasi Hills, RB: Ri Bhoi, JH: Jaintia Hills; *Denotes significance (p < 0.05) in a comparison of age group 15+ versus children (< 5 years, 5–14 years) in 2015 versus 2017. **b** The proportion of males among each age group for each year is shown for each district. *Denotes proportion of males in 15+ years group is significantly higher compared to males in 5–14 years age group or males in < 5 years age group; ^†^Denotes proportion of males in 15+ years group is significantly higher compared to males in < 5 years age group but not to males in 5–14 years age group; ^‡^Denotes proportion of males in 15+ years group is significantly less compared to males in 5–14 years age group and to males in < 5 age years age group; The Z-axis represents the API in both panels

A dramatic reduction in malaria cases in West and East Garo Hills, Ri Bhoi, and Jaintia Hills occurred in 2016, the same year that the first distribution of LLINs occurred (Table 1: reductions in reported malaria of 56.2%, 45.2%, 56.4%, and 23.8% respectively relative to 2015), and continuing in 2017 (again relative to 2015, reductions in reported malaria of 85.6%, 84.7%, 92.1%, and 64.5% respectively) as evident by the plotted data (Fig. 4). These reductions were also reflected in the API (Fig. 5). In West Khasi Hills, the decline in reported malaria occurred mainly in 2017 (e.g., reduction of 60.8% relative to 2015; Fig. 4). No reduction was noted in South Garo Hills in the years examined. Compared to 2015, the 2017 reported proportion of persons ≥ 15 years of age with malaria significantly increased in all districts except for South Garo Hills (Fig. 5a and Table 1; West Garo Hills from 56.1% in 2015 to 64% in 2017, p < 0.001; East Garo Hills 42.1–47.2%, p < 0.001; South Garo Hills 47.1% in 2015 and 2017, p = 0.670, West Khasi Hills 54.4–64.1%, p < 0.001; Ri Bhoi 48.7–59.1%, p = 0.006; and Jaintia Hills 51–64.2%, p < 0.001). Gender differences among age groups were not significant in West Khasi Hills, Ri Bhoi, and Jaintia Hills, but persisted in 2017 in the other districts in the same direction as 2015 (Fig. 5b, e.g. West Garo Hills 56.7% males in age group ≥ 15 years compared to 50.4% males in age group < 15 years, p = 0.017, East Garo Hills 55.5% female among ≥ 15 years compared to 49.5% females in age group < 15 years, p = 0.001 and South Garo Hills 57.4% female among ≥ 15 years and 50.9% in age group < 15 years, p < 0.001).

## Discussion/conclusions

This is the first systematic review of malaria epidemiology and transmission in the northeastern state of Meghalaya. In line with the Indian mainland [34], a review of the available literature on malaria in Meghalaya confirms an overall theme: it is a complex disease which has many different factors combining to ensure continued transmission, making control and prevention challenging. While *P. falciparum* malaria certainly dominates, both *P. vivax* and mixed infections are common, and severe, unusual, and fatal cases have all been reported in recent years [11, 13, 14, 19, 20]. The overwhelming diversity of key, emerging, and potential mosquito vectors, including *An. minimus*, *An. baimaii*, *An. culicifacies*, *An. annularis*, and *An. fluviatilis* amongst several others [21–25], only adds to the complexity of malaria transmission and epidemiology across the state. These reports highlight the need for continued surveillance and increased focus on both prevention methods and early diagnosis.

A second theme from the malaria in Meghalaya literature was that most studies were focused on a single district or region [11, 20] making them difficult to generalize to the state at large. For example, the study by Sharma et al. [13] reported a low *P. vivax* burden in the state of Meghalaya in comparison to other northeastern states. However, the study sites were located in the West Garo Hills district, which is known for dominant *P. falciparum* malaria and also geographically distant from the districts in Meghalaya known to harbor higher numbers of *P. vivax* malaria cases (e.g., Jaintia Hills, Ri Bhoi). Many of the vector reports from the state were also targeted to specific regions [11, 22, 26]. Taken together, the literature covering malaria epidemiology and transmission in Meghalaya is piecemeal at best. While it is clear that malaria in Meghalaya is complex, the differences in *Plasmodium* species prevalence and distribution, land use and ecology, and vector species across the state make it challenging to generalize trends from the existing epidemiology and vector literature alone.

Malaria prevention and control methods in Meghalaya are also diverse in nature and seemingly widespread across the state. Distributed interventions such as IRS, impregnated bed nets, and education/awareness programmes are commonly employed, although poor uptake of certain interventions (e.g., IRS) was reported [31]. However, the malaria prevention and control literature lacks studies that determine and characterize the effectiveness of interventions and control methods in use in Meghalaya. Such studies will become more important as malaria numbers decrease, requiring focused, context-specific efforts (e.g., village by village, region by region) to obtain gains until elimination is achieved.

While *An. minimus* and *An. baimaii* are likely to continue playing the major role in malaria transmission in Meghalaya in the forseeable future, landcover change appears to be increasing the complexity of malaria epidemiology in this region. To achieve malaria elimination, characterization of all relevant vectors and their varying ecologies and behaviours is essential if interventions are to be correctly targetted and ultimately effective. Furthermore, heightened awareness of the potential for mosquito adaptation to malaria interventions (e.g., switch to earlier biting times before bed net use as recorded for several *Anopheles* species [35]; greater exophily [36]), specifically the rollout of LLINs [37] in Meghalaya, is also key to gaining and maintaining vector control in this region. Overall, a fuller understanding of vector complexity and malaria transmission dynamics is essential for the integrated vector management approach that will likely be required for eventual malaria elimination [38].

Analysis and visual presentation of the Meghalaya State Malaria Control Programme data from 2015 to 2017 highlights the reductions in malaria cases and fatalities in five of the six districts analysed since the distribution of > 900,000 LLINs across the state. While these data are highly promising, it is not possible to determine if the introduction of LLINs directly caused the reduction in malaria cases or in combination with other control measures or factors, and further studies are required to evaluate their efficacy. Asymptomatic malaria rates and distribution, changes in vector and parasite species prevalence and distributions, and the emergence or expansion of drug resistant vector populations are all important and relevant factors requiring study. Interestingly, a significant reduction in proportion of infected children relative to adults in 2017 (post-LLIN distribution) relative to 2015 (pre-LLIN distribution) across 5 of the 6 districts analysed might suggest that children were given priority within a family dwelling for LLIN coverage. The one exception to the reduction in malaria cases seen during 2016–2017 is South Garo Hills, which had consistently high numbers of cases during the 2016 and 2017 monsoon seasons. Many of the villages in South Garo Hills are difficult to reach by car, and regions along the Meghalaya–Bangladesh border are areas that experience civil unrest. In combination, these features make the distribution of interventions such as LLINs and education/awareness programmes challenging, and may have resulted in poor distribution or use of LLINs in 2016.

Overall, the paucity of comprehensive articles on malaria in Meghalaya makes it difficult to develop a complete picture of the epidemiology, transmission and control of the disease within the state. Indeed, the regional differences in API, *Plasmodium* species prevalence and distribution, population demographics (sex, gender), and malaria-associated fatality rates from 3 years of surveillance data highlights the heterogeneous nature of malaria in Meghalaya, and underscores how generalizing information from one district or region of Meghalaya to another is not recommended. District-wise surveillance of malaria in Meghalaya as conducted by the Meghalaya State Malaria Control Programme is essential to understanding malaria epidemiology statewide and for designing and conducting studies to improve surveillance, intervention efficacy, and disease management. While it is important to consider the limitations of surveillance data and state level reporting (e.g. under/over reporting; missing districts), continued monitoring—including both active and passive surveillance—and routine follow up at the local level is required to ensure the accuracy of reported data and consistency across districts in order to maximize the impact of downstream prevention and control efforts.

## Abbreviations

ACT: artemisinin-based combination therapy
API: annual parasite index
ASHA: accredited social health activist
DDT: dichlorodiphenyltrichloroethane
DPPH: density per person hour
ITNs: insecticide-treated nets
IRS: indoor residual spray
LLINs: long-lasting insecticide-treated bed nets
LULC: land use and land cover
NVBDCP: National Vector Borne Disease Control Programme
PHC: primary health centre
PTND: per trap night density
SES: socioeconomic status
